# Supplementary material for: An observational study of intensivists’ expectations and effects of fluid boluses in critically ill patients
Source: PLoS One. 2022 Mar 24;17(3):e0265770. doi: 10.1371/journal.pone.0265770 (PMC8947412; doi:10.1371/journal.pone.0265770)
Supplement: S1 Appendix — (DOCX) [file pone.0265770.s002.docx]

**Study Questionnaire**


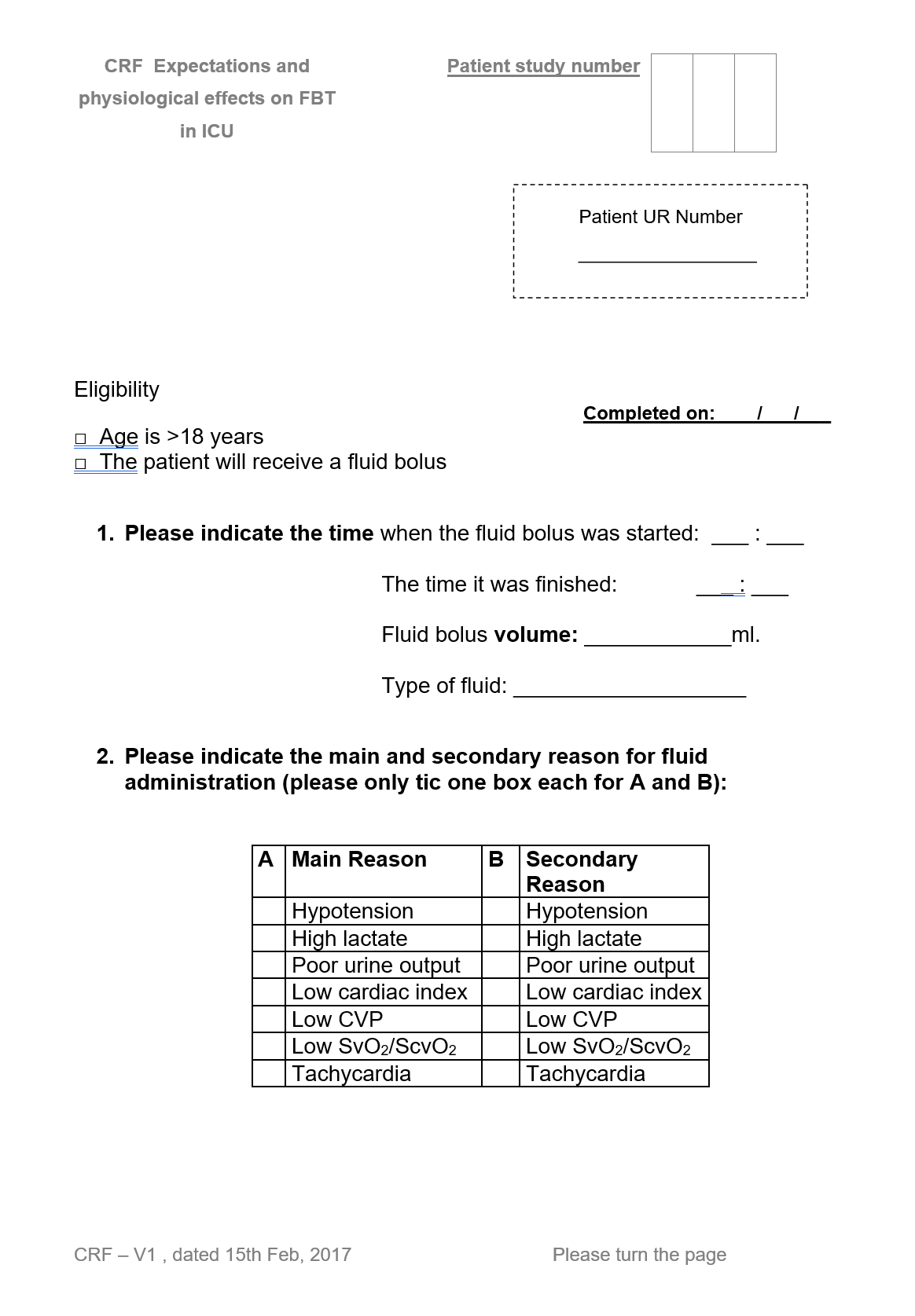


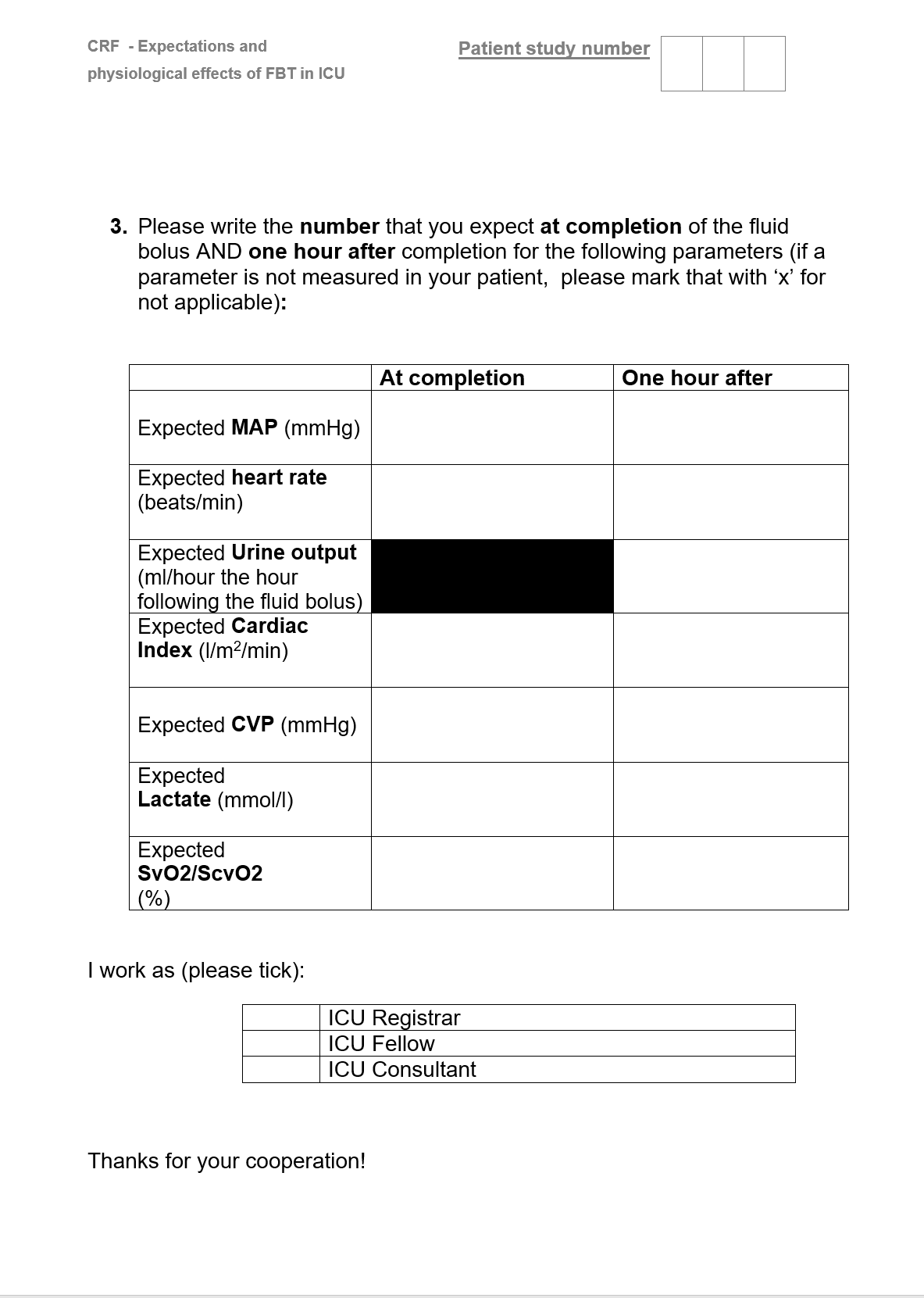


**S1 Table. Expectation for main trigger for fluid bolus**

Values are presented as median with (IQR)

| Reason for bolus | Expectations, median (IQR) | |
| --- | --- | --- |
|  | After bolus | One hour after bolus |
| Hypotension (mmHg) | +3.0 (-3.5 - 8.4) | +1.3 (-3.9 – 4.0) |
| High lactate (mmol/L) | Too small sample size | Too small sample size |
| Oliguria (ml) | N/A | +5.0 (-10.0 - 30.0) |
| Low CI (L/min/m^2^) | +0.28 (-1.75 - 0.40) | +0.08 (-1.80 - 0.40) |
| Low CVP (mmHg) | Too small sample size | Too small sample size |
| Tachycardia (bpm) | -12.4 (-14.7 - -7.3) | -8.7 (-18.8 - -5.9) |
| Low ScvO2 (%) | Too small sample size | Too small sample size |

MAP= Mean arterial pressure. UO= Urine output. HR= Heart rate. CI= Cardiac index. CVP=Central venous pressure. ScvO2= Central venous oxygen saturation.

**S2 Table. Difference between intensivist’s expectation and the physiological (effect) outcome of the FBT**

Values are presented as medians with (IQR)

| Parameter | Median absolute difference expectation/outcome after bolus (IQR) | Parameter | Median absolute difference expectation/outcome after 1 hour (IQR) |
| --- | --- | --- | --- |
| MAP (N=72) in mmHg | 6.8 (3.3-12.0) | MAP (N=72) | 5.1 (2.0-12.6) |
| UO |  | UO (N=61) | 25.0 (10.0-59.5) |
| HR (N=73) beats/min | 4.0 (1.2-10.0) | HR (N=74) | 6.9 (2.2-12.8) |
| CI (N=23) L/min/m2 | 0.2 (0.1-0.5) | CI (N=22) | 0.4 (0.2-0.6) |
| Lactate (N=22) mmol/L | 0.4 (0.2-1.0) | Lactate (N=33) | 0.4 (0.2-0.7) |
| CVP (N=21) mmHg | 3.3 (0.8-6.3) | CVP (N=21) | 2.1 (1.0-3.8) |
| ScvO2 (N=3) mmHg | Too small sample size | ScvO2 (N=3) | Too small sample size |

IQR=interquartile range; MAP= Mean arterial pressure. UO= Urine output. HR= Heart rate. CI= Cardiac index. CVP= Central venous pressure. ScvO2= Central venous oxygen saturation. N/A = Not applicable. Values are absolute difference from measured value, ie positive and negative differences are treated the same

**S3 Table. Correction for noradrenaline and propofol**

Values are presented as medians with (IQR)

| Parameter | Median value (IQR) | Median difference from uncorrected (IQR) | p-value |
| --- | --- | --- | --- |
| MAP after bolus | 71.9 (67.4-83.7) | 0.0 (0.0-0.0) | 0.72 |
| MAP after 1 hour | 73.4 (66.8-84.9) | 0.0 (0.0-0.0) | 0.49 |
| UO after 1 hour | 59.2 (26.2-100.6) | 0.0 (0.0-0.0) | 0.73 |
| HR after bolus | 90.8 (78.2-111.1) | 0.0 (0.0-0.0) | 0.88 |
| HR after 1 hour | 91.5 (80.1-111.8) | 0.0 (0.0-0.0) | 0.86 |
| CI after bolus | 2.7 (2.2-2.9) | 0.0 (0.0-0.0) | 0.00 |
| CI after 1 hour | 2.8 (2.3-3.1) | 0.0 (0.0-0.0) | 0.16 |
| Lactate after bolus | 1.5 (0.9-2.3) | 0.0 (0.0-0.0) | 0.22 |
| Lactate after 1 hour | 1.20 (0.9-2.1) | 0.0 (0.0-0.0) | 0.93 |
| CVP after bolus | 13.5 (11.6-18.3) | 0.0 (0.0-0.0) | 0.77 |
| CVP after 1 hour | 10.8 (9.0-12.3) | 0.0 (-0.4-0.0) | 0.04 |
| ScvO2 after bolus | Too small sample size | Too small sample size | Too small sample size |
| ScvO2 after 1 hour | Too small sample size | Too small sample size | Too small sample size |

IQR = interquartile range; MAP= Mean arterial pressure. UO= Urine output. HR= Heart rate. CI= Cardiac index. CVP=Central venous pressure. ScvO2= Central venous oxygen saturation

**S1 Fig. Scatter plot of measured and expected MAP one hour after fluid bolus**


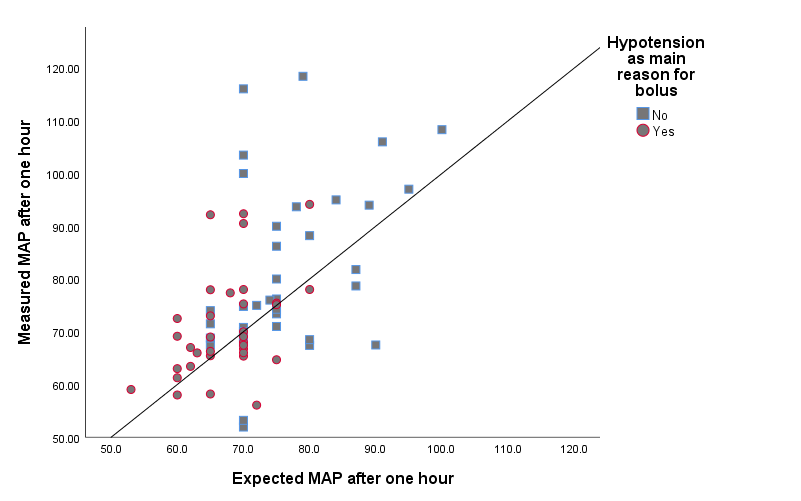


Scatter plot comparing measured and expected MAP one hour after fluid bolus. Line represents perfect fit. MAP= Mean arterial pressure

**S2 Fig. Scatter plot of measured and expected HR after fluid bolus**
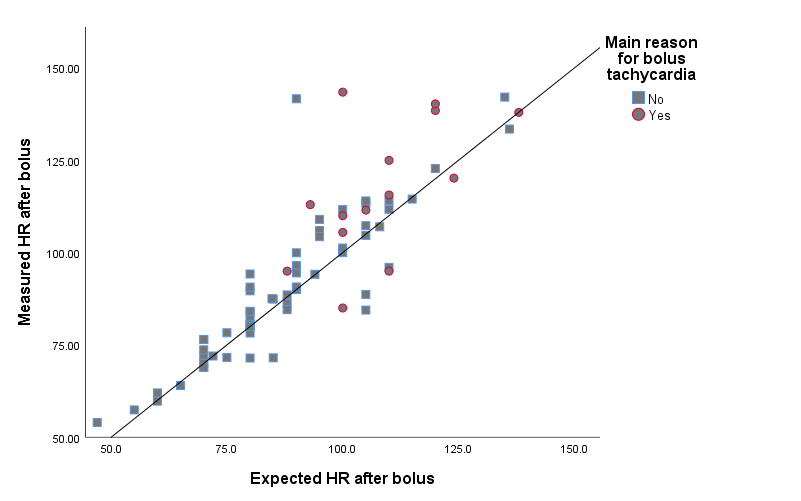


Scatter plot comparing measured and expected HR after fluid bolus. Line represents perfect fit. HR= Heart rate

**S3 Fig. Scatter plot of measured and expected HR one hour after fluid bolus**


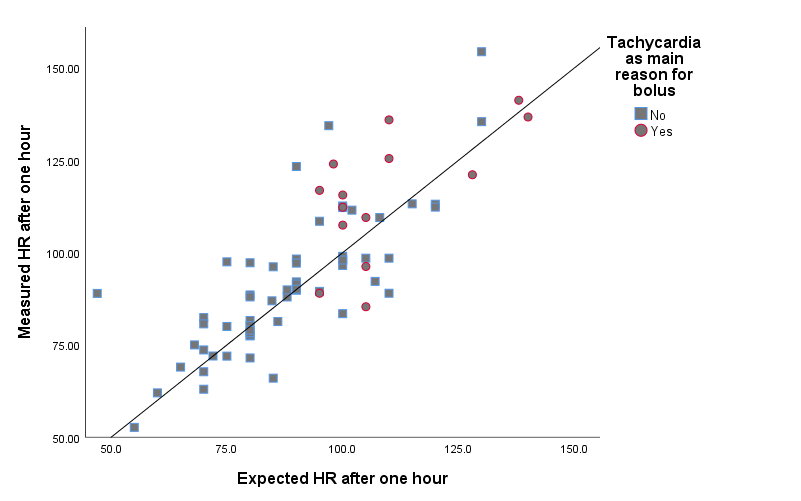


Scatter plot comparing measured and expected HR one hour after fluid bolus. Line represents perfect fit. HR= Heart rate

**S4 Fig. Adjusted Bland-Altman plot of estimated and measured MAP after fluid bolus**
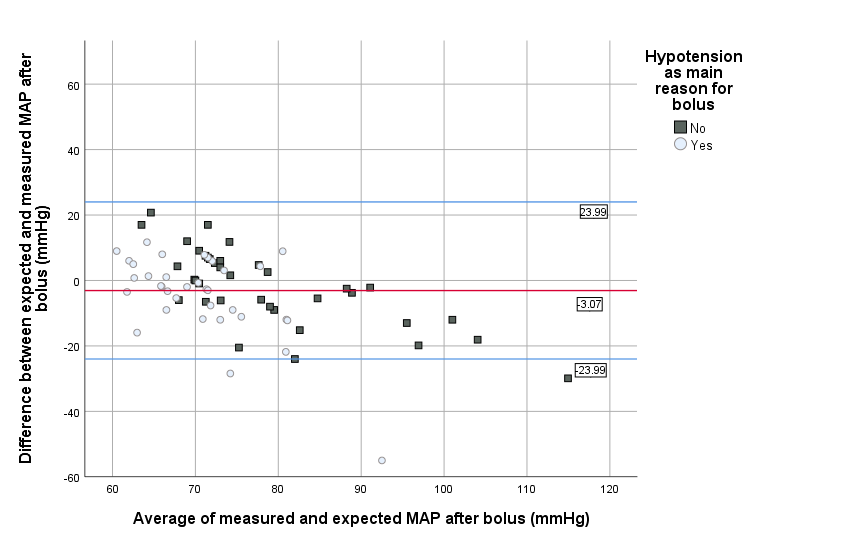


Bland-Altman plot comparing the estimated MAP against the measured MAP after fluid bolus. Limits of agreements are set to ±1.96 SD. Positive values indicate a higher value for expected MAP compared to measured, and the inverse for negative values. Red line displays mean difference between estimated and measured MAP. Blue lines display limits of agreement. MAP= Mean arterial pressure. The mean bias for expectations was toward overestimations

**S5 Fig. Adjusted Bland-Altman plot of estimated and measured MAP one hour after fluid bolus**
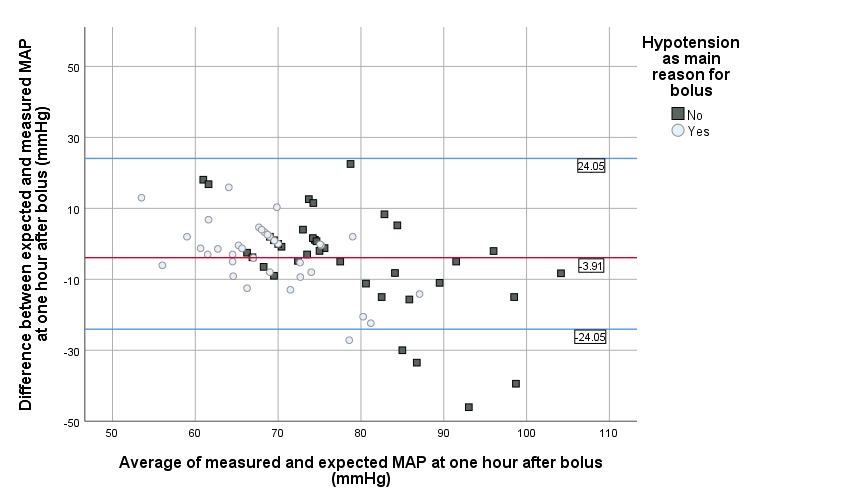


Bland-Altman plot comparing the estimated MAP against the measured MAP one hour after fluid bolus. Limits of agreements are set to ±1.96 SD. Positive values indicate a higher value for expected MAP compared to measured, and the inverse for negative values. Red line displays mean difference between estimated and measured MAP. Blue lines display limits of agreement. MAP= Mean arterial pressure. The mean bias for expectations was toward overestimations

**S6 Fig. Adjusted Bland-Altman plot of estimated and measured HR after fluid bolus**


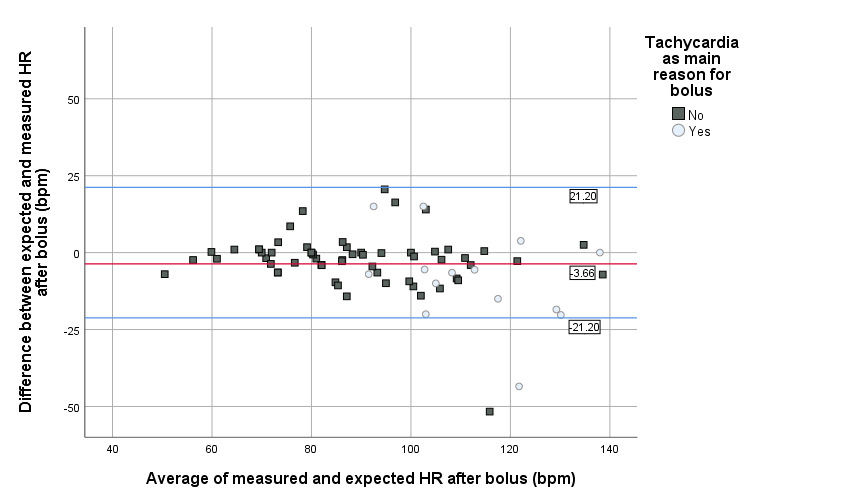


Bland-Altman plot comparing the estimated HR against the measured HR after fluid bolus. Limits of agreements are set to ±1.96 SD. Positive values indicate a higher value for expected HR compared to measured, and the inverse for negative values. Red line displays mean difference between estimated and measured HR. Blue lines display limits of agreement. HR= Heart rate. The mean bias for expectations was toward underestimation

**S7 Fig. Adjusted Bland-Altman plot of estimated and measured HR one hour after fluid bolus**


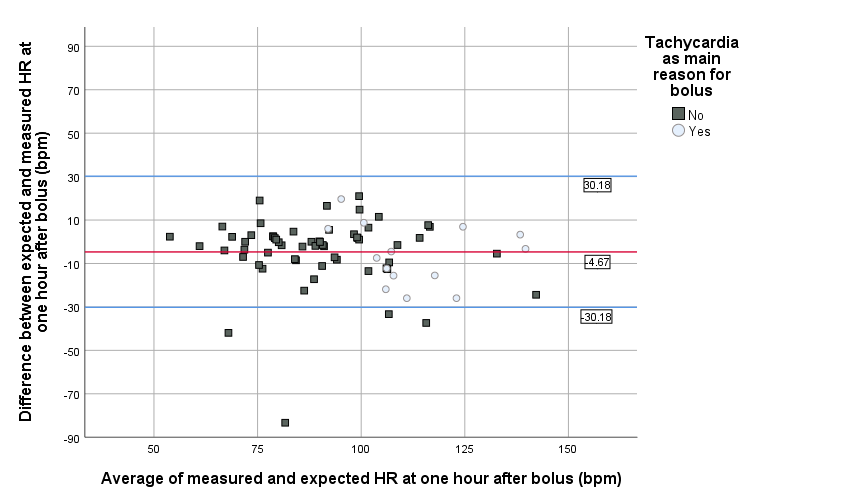


Bland-Altman plot comparing the estimated HR against the measured HR one hour after fluid bolus. Limits of agreements are set to ±1.96 SD. Positive values indicate a higher value for expected HR compared to measured, and the inverse for negative values. Red line displays mean difference between estimated and measured HR. Blue lines display limits of agreement. HR= Heart rate. The mean bias for expectations was toward underestimation

**S8 Fig. Adjusted Bland-Altman plot of estimated and measured UO one hour after fluid bolus**
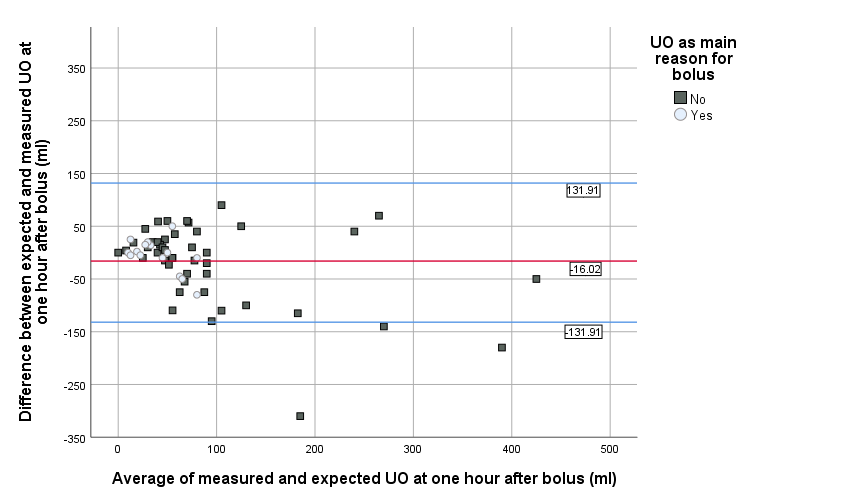


Bland-Altman plot comparing the estimated UO against the measured UO one hour after fluid bolus. Limits of agreements are set to ±1.96 SD. Positive values indicate a higher value for expected UO compared to measured, and the inverse for negative values. Red line displays mean difference between estimated and measured UO. Blue lines display limits of agreement. UO= Urine output. The mean bias for expectations was toward overestimations
