# Supplementary material for: An observational study of intensivists’ expectations and effects of fluid boluses in critically ill patients
Source: PLoS One. 2022 Mar 24;17(3):e0265770. doi: 10.1371/journal.pone.0265770 (PMC8947412; doi:10.1371/journal.pone.0265770)
Supplement: S1 Protocol — (DOCX) [file pone.0265770.s003.docx]

A prospective observational study of the expectations and the physiological effects of fluid bolus therapy in the intensive care unit

Research protocol: Version 2.6 May 2017

**Investigators**

Olof Wall, MD, Anaesthesia/ICU registrar, Danderyds Sjukhus, Stockholm. Karolinska Institutet, Department of Clinical Science and Education, Section of Anaesthesiology and Intensive Care, Södersjukhuset, Stockholm, Sweden

Eva Joelsson-Alm, RN, PhD, Karolinska Institutet, Department of Clinical Science and Education, Section of Anaesthesiology and Intensive Care, Södersjukhuset, Stockholm, Sweden

Christer Svensén, MD, PhD, Professor, Karolinska Institutet, Department of Clinical Science and Education, Section of Anaesthesiology and Intensive Care, Södersjukhuset, Stockholm, Sweden

Daniel Törnberg, MD, PhD, ICU Consultant, Danderyds Sjukhus, Stockholm, Sweden

Rinaldo Bellomo, Director of Intensive Care Research, Austin Health, VIC, Australia

Johan Mårtensson, MD, PhD, Department of Intensive Care, Austin Hospital, Melbourne, VIC, Australia

Maria Cronhjort, MD, PhD, ICU Consultant, Karolinska Institutet, Department of Clinical Science and Education, Section of Anaesthesiology and Intensive Care, Södersjukhuset, Stockholm, Sweden

**Study coordinator/contact person**

Olof Wall, Anaesthesia/ICU registrar, Danderyds Sjukhus, Stockholm. Karolinska Institutet, Department of Clinical Science and Education, Section of Anaesthesiology and Intensive Care, Södersjukhuset, Stockholm, Sweden

Department of Anaesthesia and Intensive Care, Danderyds Sjukhus, level 5, Mörbygårdsvägen, 182 88 Danderyd

Phone number: +468 123 550 00

Mobile: +4673 0736163034

Mail: olof.wall@sll.se

**Background**

Fluid therapy is probably the most ubiquitous therapeutic intervention in critically ill patients, particularly in those suffering from sepsis (1, 2). Despite this, many aspects of the intervention remain subjects of controversy. There is an ongoing discussion regarding what fluid to give, how much to give and when to give it. Furthermore, while short term goals are to improve cardiac output, systemic blood pressure and organ perfusion, the long term hemodynamic effects are not well described. Excess fluids in the intensive care unit (ICU) has been associated with increased long term morbidity (3-6). In addition, recent trials have shown potential harm due to fluid administration with an increase in mortality in pediatric septic patients in Africa (7, 8). The hemodynamic effects of fluid boluses are small and short lived (9, 10). Since fluids are currently being given in large volumes to critically ill patients in the stabilization phase without a full understanding of their physiological effects, further research in this field could have potential benefit for large numbers of patients who are at high risk of dying.

**Aim**

The aim is to investigate to what extent the expectations of the attending physician concerning hemodynamic effects from the infused fluid in the critically ill are met.

Primary outcome: To describe the accuracy and precision of the expectations of the clinicians of the effect of a fluid bolus at completion of the fluid bolus. Secondary outcomes: To describe the accuracy and precision of the expectations of the clinicians of the effect of a fluid bolus one hour after the completion of the fluid bolus. The reasons for fluid administration. Changes in hemodynamic variables (Cardiac Index (CI), heart rate, blood pressure, CVP,) and lactate, central/mixed venous saturation (ScvO2/SvO2) and urinary output.

**Methods**

Observational multi-center study of fluid administration in the ICU:s at two Swedish tertiary centers (Södersjukhuset and Danderyds Sjukhus) and one Australian university hospital (Austin Hospital). Planned recruitment of 100 adult patients.

Inclusion criteria: Admission to ICU. Age 18 years or older.

Exclusion criteria: Patients in whom death is considered imminent (within 24 hours). The intensive care physician declines to participate.

Patients will receive standard care at the discretion of the treating physicians, including all measurements and monitoring. When the treating physician chooses to give a fluid bolus (defined as 250 mL or more of fluids over 30 minutes or less), the patient will be included and the physician asked to fill out a questionnaire asking for pre-defined reasons to give fluids: hypotension, tachycardia, oliguria, low central venous pressure (CVP), high lactate levels, low mixed venous oxygen saturation (SvO_2_) or central venous oxygen saturation (ScVO_2_) and low cardiac output (CO). Also, the physician will be asked to specify what hemodynamic response of these variables is expected at completion of the bolus and one hour later. The expectations and results of the first bolus after inclusion will be measured and any further boluses given to the same patient will not lead to re-inclusion and further questionnaire’s. The first bolus after inclusion will be studied, which can be at any time during the ICU-stay. For comparison, data will be extracted from the departmental documentation system (Clinisoft^®^, TakeCare^®^ or other methods) regarding MAP, pulse rate, CO, CVP, urine output and pertinent laboratory values such as lactate, SvO_2_ or ScVO_2_ and creatinine. Measurements will be registered before the onset of the fluid bolus and until 60 min after completion of the fluid bolus. If any other static or dynamic hemodynamic variables such as Pulse Pressure Variation (PPV), Stroke Volume Variation (SVV), Extravascular Lung Water (EVLW) or Stroke Volume Index (SVI) are being measured this data will be extracted as well. Any infusions of vasoactive medications and diuretics given will also be recorded and dosages registered will be accounted for as confounders for hemodynamic changes. Any fluids administered during the study period of besides those of the fluid bolus that led to inclusion will be recorded and accounted for as confounders. Fluids will be registered as colloids, crystalloids or maintenance fluids. For MAP, pulse rate and any hemodynamic variables values will be recorded according to the sampling in the electronic system. Laboratory values will be ordered at the clinicians’ discretions as usual (usually at admission and then as indicated clinically) and extracted at these intervals. Urine output will be recorded for six hours following the fluid bolus.

**Statistical analysis**

The accuracy of the physicians’ estimations of the effect of a fluid bolus will be described using Bland-Altman methodology. In addition, clinicians’ ability to predict the hemodynamic response will be assessed by calculating sensitivity, specificity, PPV, NPV and the area under the receiver operating characteristics curve. Outcomes will be compared after log transformation where appropriate. Comparisons will be made using t-test and ANOVA for repeated-measures or Wilcoxon signed-rank test and Kruskal-Wallis test according to the underlying distribution for continuous data and Fishers’s exact test for categorical data. Multiple regression analysis will also be performed adjusting for predefined confounding factors associated with hemodynamic changes: change in vasopressors, sedatives, diuretics, change in pacemaker rhythm, additional fluid boluses.

Ethical considerations

Ethical approval will be sought from the Ethical Review Board of Stockholm and the Austin ethical review board.

Information will be posted on the study sites, and if objections are raised to inclusion patients will not be included.

Patients in Stockholm will be covered by Sweden’s Patient Insurance policy.

Data collected will be coded and treated confidentially

The study will be observational and no changes will be made in the care of the patients included. There are no foreseeable risks for the patients to be included in this study.

**Preliminary results**

There are no results yet. The study is planned to be performed during fall 2017.

**Funding**

Funding will be sought from the Stockholm County Council (ALF) and external grants. A personal grant has been received for Olof Wall (Forskar-ST).

**References**

1. Finfer S, Liu B, Taylor C, Bellomo R, Billot L, Cook D, et al. Resuscitation fluid use in critically ill adults: an international cross-sectional study in 391 intensive care units. Crit Care. 2010;14(5):R185.

2. Magder S. Bench-to-bedside review: An approach to hemodynamic monitoring--Guyton at the bedside. Crit Care. 2012;16(5):236.

3. Boyd JH, Forbes J, Nakada TA, Walley KR, Russell JA. Fluid resuscitation in septic shock: a positive fluid balance and elevated central venous pressure are associated with increased mortality. Crit Care Med. 2011;39(2):259-65.

4. Murphy CV, Schramm GE, Doherty JA, Reichley RM, Gajic O, Afessa B, et al. The importance of fluid management in acute lung injury secondary to septic shock. Chest. 2009;136(1):102-9.

5. Sadaka F, Juarez M, Naydenov S, O'Brien J. Fluid resuscitation in septic shock: the effect of increasing fluid balance on mortality. J Intensive Care Med. 2014;29(4):213-7.

6. Vincent JL, Sakr Y, Sprung CL, Ranieri VM, Reinhart K, Gerlach H, et al. Sepsis in European intensive care units: results of the SOAP study. Crit Care Med. 2006;34(2):344-53.

7. Maitland K, George EC, Evans JA, Kiguli S, Olupot-Olupot P, Akech SO, et al. Exploring mechanisms of excess mortality with early fluid resuscitation: insights from the FEAST trial. BMC Med. 2013;11:68.

8. Maitland K, Kiguli S, Opoka RO, Engoru C, Olupot-Olupot P, Akech SO, et al. Mortality after fluid bolus in African children with severe infection. N Engl J Med. 2011;364(26):2483-95.

9. Glassford NJ, Eastwood GM, Bellomo R. Physiological changes after fluid bolus therapy in sepsis: a systematic review of contemporary data. Crit Care. 2014;18(6):696.

10. Bihari S, Prakash S, Bersten AD. Post Resusicitation Fluid Boluses in Severe Sepsis or Septic Shock: Prevalence and Efficacy (Price Study). Shock. 2013;40(1):28-34.
